# Mechanosensitive enteric neurons in the guinea pig gastric corpus

**DOI:** 10.3389/fncel.2015.00430

**Published:** 2015-11-03

**Authors:** Gemma Mazzuoli-Weber, Michael Schemann

**Affiliations:** Human Biology, Technische Universitaet MuenchenFreising, Germany

**Keywords:** enteric nervous system, myenteric neuron, mechanosensitivity, multifunctional

## Abstract

For long it was believed that a particular population of enteric neurons, referred to as intrinsic primary afferent neuron (IPAN)s, encodes mechanical stimulation. We recently proposed a new concept suggesting that there are in addition mechanosensitive enteric neurons (MEN) that are multifunctional. Based on firing pattern MEN behaved as rapidly, slowly, or ultra-slowly adapting RAMEN, SAMEN, or USAMEN, respectively. We aimed to validate this concept in the myenteric plexus of the gastric corpus, a region where IPANs were not identified and existence of enteric sensory neurons was even questioned. The gastric corpus is characterized by a particularly dense extrinsic sensory innervation. Neuronal activity was recorded with voltage sensitive dye imaging after deformation of ganglia by compression (intraganglionic volume injection or von Fry hair) or tension (ganglionic stretch). We demonstrated that 27% of the gastric neurons were MEN and responded to intraganglionic volume injection. Of these 73% were RAMEN, 25% SAMEN, and 2% USAMEN with a firing frequency of 1.7 (1.1/2.2), 5.1 (2.2/7.7), and of 5.4 (5.0/15.5) Hz, respectively. The responses were reproducible and stronger with increased stimulus strength. Even after adaptation another deformation evoked spike discharge again suggesting a resetting mode of the mechanoreceptors. All MEN received fast synaptic input. Fifty five percent of all MEN were cholinergic and 45% nitrergic. Responses in some MEN significantly decreased after perfusion of TTX, low Ca^++^/high Mg^++^ Krebs solution, capsaicin induced nerve defunctionalization and capsazepine indicating the involvement of TRPV1 expressing extrinsic mechanosensitive nerves. Half of gastric MEN responded to intraganglionic volume injection as well as to ganglionic stretch and 23% responded to stretch only. Tension-sensitive MEN were to a large proportion USAMEN (44%). In summary, we demonstrated for the first time compression and tension-sensitive MEN in the stomach; many of them responded to one stimulus modality only. Their proportions and the basic properties were similar to MEN previously identified by us in other intestinal region and species. Unlike in the intestine, the responsiveness of some gastric MEN is enhanced by extrinsic TRPV1 expressing visceral afferents.

## Introduction

The enteric nervous system (ENS) forms along the gastrointestinal tract a continuous network of ganglia interconnected via nerve bundles. These ganglia are made up of neurons that are able to regulate all gastrointestinal functions autonomously from the central nervous system. To function in an autonomous way, such a system requires neurons specialized to sense different stimuli. For many years prevailed the plausible concept proposing the existence of specialized neuronal populations, functionally defined as sensory, inter and motor-neurons. Such neuron populations had distinct morphologies, chemical codes and projections which mostly fitted to their functions. In the last few years we and others identified mechanosensitive enteric neurons that did not fulfill these strict criteria but had features that rather classified them as multifunctional neurons (Spencer and Smith, 2004; Mazzuoli and Schemann, 2009, 2012; Dong et al., 2015).

We previously described multifunctional mechanosensitive enteric neurons (MEN) in the guinea pig ileum (Mazzuoli and Schemann, 2009; Kugler et al., 2015), in the mouse ileum and colon (Mazzuoli and Schemann, 2012) as well as in the human intestine (Kugler et al., 2015). Depending on the region studied, 15–25% of all enteric neurons were MEN. Based on their firing pattern they behaved as rapidly, slowly, or ultra-slowly adapting neurons, RAMEN, SAMEN, or USAMEN, respectively. RAMEN made up 60 and 56% of the mechanosensitive myenteric neurons in the guinea pig and mouse ileum, respectively. The proportions of SAMEN and USAMEN were smaller and amounted to 36 and 42% in the guinea pig and mouse ileum, respectively; the group of USAMEN was with 4 and 2% the smallest. In the myenteric plexus of the guinea pig colon the proportion of MEN behaving as RAMEN, SAMEN, or USAMEN were 38, 56, or 5%; in the mouse colon the corresponding values were 41% RAMEN, 38% SAMEN, and 21% USAMEN (Mazzuoli-Weber and Schemann, 2015). In both studies we used a 400 ms intraganglionic volume injection as a mechanical stimulus (Mazzuoli and Schemann, 2009, 2012). This type of mechanical stimulus evoked in the ganglia a mixture of compressive and shearing forces. We hypothesized that the differences in firing pattern between the ileum and colon might be the more phasic or tonic muscle activity prevailing in the ileum and colon, respectively.

The classical intrinsic primary afferent neuron (IPAN)s with the electrophysiological feature of a slow after hyperpolarization (AH) are absent in the myenteric plexus of the guinea pig gastric corpus (Schemann and Wood, 1989a). Calbindin (Calb) expression as another feature of IPANs is present in only 12% of myenteric neurons in the corpus region and these were mostly located at the greater curvature (Reiche et al., 1999, 2001). The lack of AH-IPANs was for a long time interpreted as an absence of enteric sensory neurons in the stomach. It was believed that extrinsic sensory neurons fulfill this task as the stomach receives a rich innervation from nerves that originate in the nodose ganglia or in the spinal cord (Berthoud and Powley, 1992; Wang and Powley, 2000). Many of these were functionally identified as mechanosensitive nerves terminating as intraganglionic laminar endings (IGLEs) in myenteric ganglia or as intramuscular arrays (IMA) in the muscle layers (Zagorodnyuk et al., 2001). Many of these endings contain substance P (SP) (Sharkey et al., 1984; Mawe et al., 1989) which activates gastric myenteric neurons by neurokinin (NK)-3 receptors (Schemann and Kayser, 1991).

It is for the following reasons unlikely that extrinsic afferents alone sense mechanical stimulation and generate reflex activity. A number of neural reflexes in the stomach, such as the accommodation reflex, are mediated by the ENS (Paton and Vane, 1963; Desai et al., 1991; Hennig et al., 1997). In addition, distension evoked polarized enteric nerve reflexes were shown in isolated gastric preparations (Schemann et al., 2008). We hypothesize that the recently identified multifunctional MEN in the myenteric plexus of the small and large intestine might be candidate enteric sensory neurons in the stomach. Support for this concept came from the observation that some gastric myenteric neurons have multiple long processes which ramify in ganglia, muscle, and/or mucosa (Schemann and Schaaf, 1995). We already speculated at that time, that these neurons might be multifunctional. However, it remained unknown whether they respond to mechanical deformation and whether their properties are comparable to those MEN identified in the guinea pig and mouse small and large intestine.

We therefore aimed to identify and characterize MEN in the guinea pig gastric corpus, a gut region that exhibits tonic as well as phasic motor activity (Petkov and Boev, 1999) and which contains myenteric neurons with electric and synaptic properties very different from enteric neurons in the other gut regions (Schemann and Wood, 1989a,b). In order to understand if MEN respond to different stimulus modalities we exposed the gastric neurons to two different type of mechanical stimulation: intraganglionic injection of small volume of Krebs solution, causing compressive and shear stress and ganglionic stretch, evoking tensile stress. We validated the intraganglionic volume injection method performing parallel experiments with von Frey hair compression. Finally, we wanted to provide further support for the concept that multifunctional MNS, which do not belong to the AH-IPANs population, exist throughout the gut.

## Material and methods

### Ethical approval

All procedures and techniques have been previously described in details (Michel et al., 2011; Mazzuoli and Schemann, 2012).

All guinea pig work was conducted according to the German guidelines for animal care and welfare (Deutsches Tierschutzgesetz) and approved by the Bavarian state ethics committee (Regierung Oberbayern, which serves as the Institutional Care and Use Committee for the Technische Universitaet Muenchen) according to §4 and §11 Deutsches Tierschutzgesetz under the reference number 32-568-2.

### Tissue samples

Male guinea pigs “Dunkin Hartley” (Harlan GmbH, Borchen, Germany) were used. Guinea pigs (340 ± 60 g) were killed by cervical dislocation followed by exsanguination. The stomach was quickly removed and further dissected in Carbogen aerated (95% O_2_, 5% CO_2_; pH = 7.40) Krebs solution containing (in mM): 117 NaCl, 4.7 KCl, 1.2 MgCl_2_ 6 H_2_O, 1.2 NaH_2_ PO_4_, 25 NaHCO_3_, 2.5 CaCl_2_, 2 H_2_O, 11 glucose. The mucosa, the submucosa and the circular muscle layer of the corpus were gently removed in order to obtain a longitudinal muscle-myenteric plexus preparation. This preparation (5 × 10 mm) was pinned onto a silicone ring that was placed in a recording chamber continuously perfused with 37°C carbogen bubbled Krebs solution with a rate of perfusion of 11 ml/min. After mounting the tissue the average amount of stretch above slack was 29% ± 3% in the circular and 48% ± 9% in the longitudinal direction.

To block synaptic transmission 20 min perfusion of the entire tissue with a low Ca^++^/high Mg^++^ Krebs solution, containing (in mM) 98 NaCl, 4.7 KCl, 16 MgCl_2_ 6 H_2_O, 1.2 NaH_2_ PO_4_, 25 NaHCO_3_, 0.25 CaCl_2_ H_2_O, 11 glucose, was used.

### Ultra-fast neuroimaging technique

An ultra-fast neuroimaging technique coupled with a voltage sensitive dye was used. Individual ganglia were loaded with the fluorescent voltage-sensitive dye Di-8-ANEPPS (1-(3-sulfanatopropyl)-4-[beta[2-(di-n-octylamino)-6-naphthyl]vinyl] pyridinium betaine) (Invitrogen, Karlsruhe, Germany) by local application through a microejection pipette loaded with 20 μM Di-8-ANEPPS dissolved in DMSO and pluronic F-127 containing Krebs solution. This staining did not change the electrophysiological properties of the nerve cells (Neunlist et al., 1999). The chamber containing the preparation was mounted onto an inverted epifluorescence Olympus IX 71 microscope (Olympus, Hamburg, Germany) equipped with a 75-W xenon arc lamp (Optosource, Cairn Research Ltd., Faversham, UK). Illumination of the preparation was achieved by a software operated shutter (Uniblitz D122, Vincent Associates, New York, USA). The light emitted by a Xenon lamp excites Di-8-ANEPPS through a modified Cy3 fluorescence filter. This set up allowed us to measure relative changes in the fluorescence (ΔF/F), which is linearly related to changes in the membrane potential (Neunlist et al., 1999). Changes in fluorescence intensity were detected by a CCD camera (80 × 80 pixels; RedShirt Imaging, Decatur, USA) at a sampling rate of 1 kHz. The recordings were taken with a × 40 objective resulting in a spatial resolution of 29 μm^2^/pixel. The fluorescent images were acquired and processed by the Neuroplex 9.9.6 software (RedShirt Imaging).

To evoke fast EPSPs via electrical stimulation of interganglionic fiber tracts a Teflon-coated platinum electrode (diameter 20–25 μm) connected to a constant-voltage stimulator was used (600 μs pulse duration with amplitudes ranging from 1 to 8 V which were supra-threshold).

### Mechanical stimulation of ganglia and neurons

The mechanical stimulation techniques used are the intraganglionic injections of small volumes of Krebs solution, von Frey hair probing (both methods already applied successfully in the guinea pig ileum and in the mouse small and large intestine) and ganglionic stretch (Mazzuoli and Schemann, 2009, 2012).

For the intraganglionic volume injection a glass pipette filled with the same oxygenated and buffered Krebs solution that was used for superfusing the preparation was inserted into a fiber tract in the portion where it entered the ganglion. Volumes were injected by a pressure controlled picospritzer (Parker Hannifin Co., Cleveland, OH, USA) for 400 ms. The pressure was adjusted to values that led to deformation of the entire ganglion as visually inspected. As previously described, the intraganglionic volume injection caused a biphasic deformation (Mazzuoli and Schemann, 2009, 2012). An initial dynamic deformation caused by the inflow of the Krebs solution which was followed by a sustained deformation, which was due to the very slow volume redistribution. The sustained deformation lasted 120 ± 60 s. Changing the injection pressure resulted in stronger deformation.

The von Frey hair stimulation was obtained by probing the ganglia with a 35 μm diameter hair (1.25 mN) which was connected to a motorized micromanipulator (DC-3K, Märzhauser, Wetzlar, Germany) to control its advancement. The hair was placed 1–2 μm above the ganglion and then advanced to create ganglion deformation with a step size of 10 μm, then left in place for 1 min and finally retracted.

Ganglionic stretch was achieved by forcing apart two L-shaped wires which were positioned onto the tissue ~100 μm above and below a ganglion. One of the wires was connected to a motorized micromanipulator (DC-3K, Märzhauser, Wetzlar, Germany); the other wire was just placed onto the tissue. The mobile wire was moved at a speed of 150 μm/s perpendicular to the ganglion axis. This type of stimulus also caused an initial fast dynamic deformation lasting for 1234 ± 525 ms followed by a sustained deformation when the wire stayed in place to maintain tissue stretch.

This allowed us to study responses to different kinds of mechanical stresses. While stretch caused elongation and thereby a tensile stress, intraganglionic injection evoked a mixture of compressive and shearing forces.

Preliminary experiments to ensure reproducibility of the responses were performed repeating the stimulations at least three times at 5–10 min intervals. Moreover, after fixation of the tissue, any possible cell damage could be excluded by immunohistochemical staining for Hu C/D neuronal protein antibody that allowed us to check for intact cellular morphology.

### Pharmacology

Different substances were added to the Krebs solution perfusing the tissue for pharmacological studies. One micrometer tetrodotoxin (TTX) was used to block the voltage-gated Na^+^ channels. As previously reported we blocked glia response evoked by mechanical stimulation by 2 μM of the phospholipase C blocker U73122 (Zhang et al., 2003). Ten micrometer capsaicin (Sigma, Schnelldorf, Germany) was used to defunctonalize extrinsic afferents (Weber et al., 2001). Ten micrometer capsazepine (Biotrend, Cologne, Germany) and 1 μM SR142801 (Sanofi, Frankfurt, Germany) were also used. At this concentration capsazepine has been shown to be an effective and reversible transient receptor potential vanilloid receptor 1 (TRPV1) antagonist (Schicho et al., 2006). SR142801 is neurokinin (NK)3 receptor antagonist.

Before adding the substances two control mechanical stimulations were performed. Then, the antagonists were added to the perfusing Krebs solution for 25 min and another mechanical stimulation was performed. When the substance had a reversible effect, another mechanical stimulus was applied after 30 min wash out.

### Immunohistochemistry

Tissue specimens were fixed overnight at room temperature in a solution containing 4% paraformaldehyde and 0.2% picric acid in 0.1 mol/l phosphate buffer and then washed (3 × 10 min) in phosphate buffer. The preparations were first incubated in phosphate buffered saline (PBS)/NaN3 (0.1%)/horse serum (HS, 4%) for 1 h at room temperature followed by 48 h and 12 h incubation with the primary and secondary antibody, respectively. As primary antibodies we used Hu C/D neuronal protein antibody biotin conjugated (1:100; A21272; Life Technologies, Darmstadt, Germany), goat anti choline acetyltransferase (ChAT; 1:100; AB144P; Chemicon, Limburg, Germany), rabbit anti nitric oxide synthase (NOS; 1:500; 210-501-R025; Alexis, Gruenberg, Germany), rabbit anti-Calb (1:1000; AB1778; Chemicon) and rabbit anti transient receptor potential vanilloid (TRPV)1 (1:2000; ACC-030; Alomone labs, Jerusalem Israel). As secondary antibodies we used donkey anti-rabbit conjugated to 7-amino-4-indodicarbocyanin (Cy5; 1:200; 711 175 152; Dianova; Hamburg, Germany), streptavidin conjugated to dichlorotriazinylaminofluorescein (DTAF; 1:200; 016 010 084; Dianova), donkey anti goat Cy5 (1:500; 705 175 147; Dianova), and donkey anti rabbit conjugated to CF 488 (Cy2; 1:200; 711-225-152; Dianova).

Finally, specimens were washed in PBS, mounted on poly-l-lysine-coated slides and cover slipped with a solution of PBS (pH 7.0)/NaN_3_ (0.1) containing 65% glycerol. The preparations were examined with an epifluorescence microscope (Olympus), equipped with appropriate filter blocks. Images were acquired with a video camera (Fluoview 2, Olympus) connected to a computer and controlled by the “Analysis” software (Olympus). Frame integration and contrast enhancement were employed for image processing. Besides specifications provided by the suppliers, specificity of primary antibodies were previously published for rabbit anti NOS (Kummer et al., 1992), goat anti ChAT (Li and Furness, 1998; Pfannkuche et al., 1998b), and rabbit anti Calb (Reiche et al., 1999).

### Data analysis and statistic

We counted the number of labeled neurons in each ganglion and we analyzed the number of mechanosensitive neurons per ganglion and the frequency of their action potentials discharge. In addition the duration of spike discharge was calculated. We additionally analyzed an adaptation index (AI) of the mechanosensitive neurons with the following equation (Kugler et al., 2015):

$$\begin{array}{l} \text{AI}=\frac{\text{action potential frequency }500\text{ms}-\text{end of the recording}}{\text{action potential frequency }0-500\text{ms}} \end{array}$$

According to their firing behavior we could distinguish three populations, RAMEN, SAMEN, and USAMEN (Kugler et al., 2015). The first one is made up of neurons which fire action potentials only during the first 500 ms after the beginning of the deformation and show a rapid adaptation (AI = 0). These neurons, according with our previous findings, were defined RAMEN (Mazzuoli and Schemann, 2009, 2012). The second population is made up of neurons having an AI > 0 and < 1, they were firing action potentials also after the first 500 ms after the stimulus onset but the firing rate is decreasing along the recording time. These neurons, also according to our previously published works were named SAMEN. The third population had an AI ≥ 1. These neurons keep firing along the recording without any decrease of the action potential frequency. They were named USAMEN.

We calculated the changes in the surface area of a ganglion before and after application of the intraganglionic volume injection or ganglionic stretch at the time of maximal deformation as previously published (Mazzuoli and Schemann, 2012).

For signal and image analysis we used Neuroplex 10.1.2 (RedShirt Imaging), Igor Pro 6.22A (Wavemetrics Inc., Lake Oswego, OR, USA) and Image J 1.43u (Wayne Rasband, National Institute of Health, USA) software. For movie editing the program Lightworks (EditShare EMEA, UK) was used. The statistical analyses and graphics were performed with Sigmaplot 12.5 (Systat Software Inc., Erkrath, Germany). Data are presented as mean ± standard deviation or, when not normally distributed, as median values together with the 25 and 75% quartiles given in brackets. Differences in the spike frequency between several control stimulations were performed with a One-way repeated measures analysis of variance (ANOVA) test. Action potential frequency in the same neuron and number of responding neurons in the same ganglion after 2 subsequent stimuli were tested with a paired *t*-test. To test for differences in action potential frequency before and after blockade of synaptic transmission, or pharmacological treatment we used Wilcoxon Signed Rank Test since most of the data were not normally distributed. Differences were considered significant when *P* < 0.05.

## Results

### MEN responding to intraganglionic volume injection

**Figure 1:**
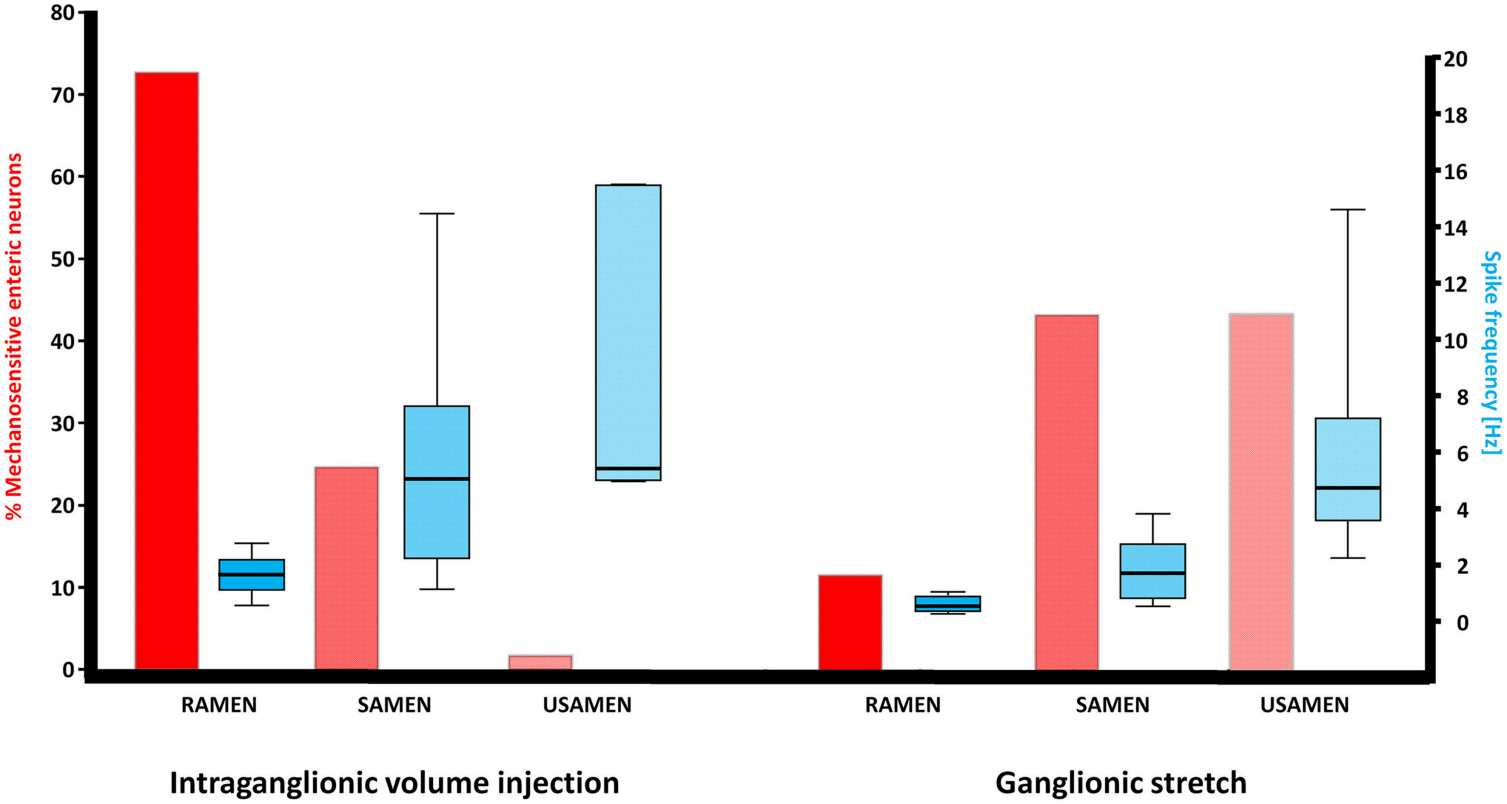
**Illustration of the neuronal response patterns after intraganglionic volume injection and ganglionic stretch**. While MEN responding to intraganglionic volume injection were mainly RAMEN, MEN responding to ganglionic stretch were significantly more often SAMEN or USAMEN, i.e., their spike discharge adapted more slowly. The spike frequency gradually increased with the increase in adaptation but this pattern was comparable between MEN responding to intraganglionic volume injection or ganglionic stretch.

Intraganglionic volume injection was performed in 40 ganglia from 23 guinea pigs. Of all neurons in a given ganglion, 27 ± 15% were mechanosensitive. The dynamic deformation lasted for 469 ± 179 ms. The flow rate was 502 ± 69 μm/s. The degree of ganglion deformation expressed as percentage change in the ganglionic area after intraganglionic volume injection was with 0.2 (0.0/0.7)% almost negligible. In the myenteric plexus of the gastric corpus 73% of the mechanosensitive neurons were identified as RAMEN, 25% as SAMEN, and 2% as USAMEN (Figure 1). The overall firing frequency during the 2 s of recording was of 1.7 (1.1/2.2), 5.1 (2.2/7.7), and of 5.4 (5.0/15.5) Hz for RAMEN, SAMEN, and USAMEN, respectively (Figure 1). These results were reproducible as the responses to a second stimulus 10 min later produced almost identical results: 76% RAMEN, 23% SAMEN, 1% USAMEN with a firing frequency of 1.6 (1.1/1.7), 4.8 (2.1/6.8), and 5.0 (4.7/12) Hz, respectively. An example of RAMEN and SAMEN firing in response to intraganglionic volume injection is shown in Supplementary Material Movie 1.

In 25 ganglia we counted a total number of 17 spontaneously active cells. In 11 of them the action potential frequency was increased after ganglionic stretch. In none of them the spike frequency was decreased by the mechanical stimulus.

**Figure 2:**
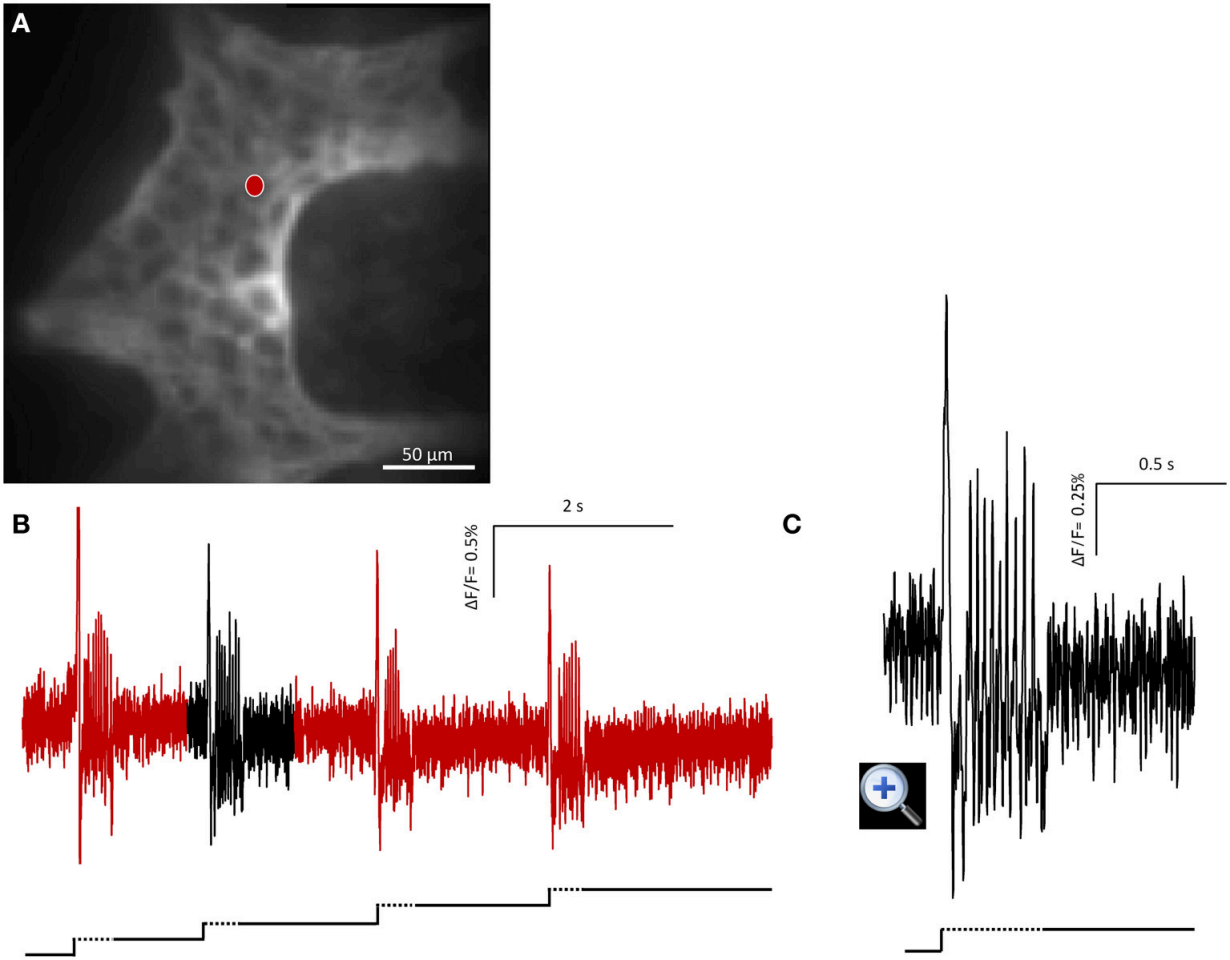
**Mechanosensitive enteric neurons (MEN) responded to repeated intraganglionic volume injections despite the fact that already the first volume injection induced a deformation maintained through the 8 s recording period**. The neuron fired every time an additional injection was applied. This finding demonstrates that the rapid adaptation was not due to desensitization of the mechanosensor but rather the result of resetting the threshold. **(A)** Shows the image of a gastric myenteric ganglion taken by the CCD camera. One MEN is marked with a red dot. The corresponding trace in response to four repeated stimulations is shown in **(B)**. The broken and solid lines beneath the traces illustrate the fast dynamic and sustained deformation during volume injection, respectively. Note that each injection caused an additional deformation but evoked similar spike discharge. The response marked in black is expanded in **(C)**.

In order to check whether the adaptation in firing is caused by desensitization we performed four intraganglionic volume injections during 8 s recording periods (seven ganglia from three guinea pigs). It has to be noted that this meant additional deformation with each injection as there was not sufficient time for volume redistribution to regain the original ganglion shape. Despite this, the firing pattern remain similar and frequency was not significantly different between the four stimulations (*p* = 0.36): I stimulation 1.4 (0.1/2.6) Hz, II stimulation 1.4 (0.7/4.3) Hz, III stimulation 2.0 (0.7/3.8) Hz, and IV stimulation 2.0 (0.7/5.1) Hz (Figure 2).

As outlined in the discussion we suggest that the compressive stress evoked during intraganglionic volume injection is more relevant than the shear stress.

We performed experiments with gradual increase in injection pressure set at 40, 60, and 100 and KPa (in a random order, 14 ganglia in four guinea pigs). These pressures were set at the gauge and we cannot comment on the actual intraganglionic pressure changes. The percentage of mechanosensitive neurons gradually increased from 0 (0.0/10.1)%, 10.7 (3.0/21.4)% to 19.4 (12.7/33.3)%. The spike discharge also increased with increasing pressure (28 neurons): 0.6 (0.0/3.2), 3.6 (1.7/5.5), and 5.2 (2.3/7.6) Hz. The enhanced spike frequency was associated with a slower adaptation reflected by the change in RAMEN:SAMEN:USAMEN proportion: with 40 Kpa 87.5% of the neurons were RAMEN and 12.5% SAMEN, with 60 Kpa 78% RAMEN and 22% SAMEN and with 100 Kpa 60% RAMEN, 36% SAMEN and 4% USAMEN.

In 12 ganglia from six guinea pigs we perfused the entire tissue with low Ca^++^/high Mg^++^ Krebs solution. After all fast EPSPs were blocked we performed another mechanical stimulation and found that the proportion of mechanosensitive neurons was significantly reduced from a median of 22 (12/29)% to 14 (7/27)% per ganglion (*p* = 0.016). The overall firing frequency of those 48 neurons which still responded in the presence of low Ca^++^/high Mg^++^ Krebs solution was not significantly different: 1.7 (1.0/3.0) Hz vs. 1.3 (0.5/2.8) Hz. Before and after synaptic blockade the proportion of RAMEN, SAMEN, and USAMEN remained with 75, 17, and 8% the same.

We previously showed that action potentials of myenteric neurons in the gastric corpus evoked by intracellular current injection were exclusively carried by TTX-sensitive sodium influx (Schemann and Wood, 1989a). It was therefore expected to find that TTX reduced spike discharge but unexpected that it didn't abolish mechanosensitive responses (Five ganglia from four guinea pigs). The percentage of MEN decreased in TTX from 33 ± 12 to 9 ± 3% (*p* = 0.02; five ganglia, four guinea pigs). The overall spike frequency of the 15 neurons that still fired in TTX was also significantly lowered by the toxin from 1.7 (1.1/2.8) to 1.1 (0.6/1.1) Hz. The neurons still responding in TTX were 88% RAMEN, 6% SAMEN, and 6% USAMEN.

It has been shown that mechanical stimulation of glia was critically dependent on phospholipase C (Zhang et al., 2003). Perfusion of the phospholipase C blocker U73122 did neither change the proportion of MEN (29 ± 7 vs. 29 ± 7%) nor the spike frequency (1.7 (1.1/2.8) Hz vs. 1.1 (1.1/3.3) Hz (6 ganglia from 2 guinea pigs).

### Occurrence of fast EPSPs in MEN

Fast EPSPs were recorded without exceptions from all neurons responding to the mechanical stimuli. Their amplitude was 0.6 (0.3/1.0) ΔF/F. After perfusion with low Ca^++^/high Mg^++^ Krebs solution all fast EPSPs were completely abolished.

### MEN responding to intraganglionic volume injection and von Frey hair probing

**Figure 3:**
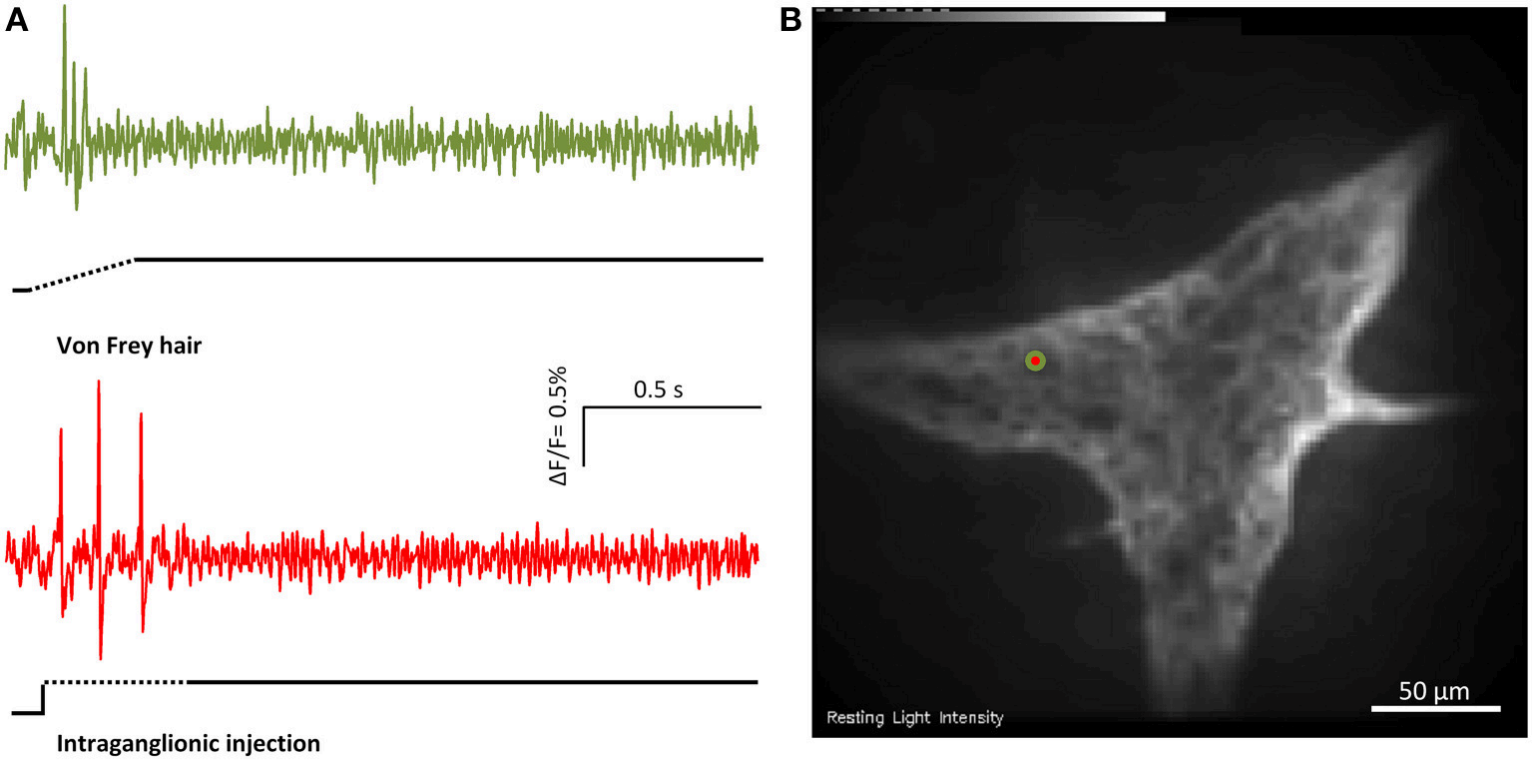
**Von Frey hair probing and and intraganglionic volume injection evoked similar responses**. **(A)** Shows the responses of the marked MEN (red/green dot in **B**) to von Frey hair probing (green trace) or intraganglionic volume injection (red trace) the two compression stimuli.

Intraganglionic volume injection is not widely used as a mechanical stimulus. We therefore performed parallel experiments using intraganglionic volume injection and the well-established von Frey hair probing (Figure 3). While the intraganglionic volume injection was deforming all cells in a given ganglion, the von Frey hair was only deforming a portion of the ganglion. Considering the neurons that were deformed by both stimuli 95 ± 2% responded to intraganglionic volume injection and to von Frey hair probing (six ganglia). 92 ± 2% of the MEN identified by von Frey hair probing also responded to the intraganglionic volume injection. The reason that there was no 100% overlap was very likely related to the different stimulus strengths. In response to von Frey hair probing 57% of the responding neurons were RAMEN and 43% were SAMEN.

### MEN responding to ganglionic stretch (tensile stress)

We performed stretch experiments in 14 ganglia from four guinea pigs. The degree of deformation expressed as percentage change in the ganglionic area after stretch was 3.1 [0.8/3.9]% and much larger than after intraganglionic volume injection (*p* = 0.002). We identify 27 ± 14 neurons per ganglion responding to ganglionic stretch. From these neurons 12% were defined as RAMEN, 44% as SAMEN, and 44% as USAMEN (Figure 1). The firing frequency during the 4 s recordings was for the RAMEN 0.5 (0.3/0.9) Hz, for the SAMEN 1.7 (0.8/2.8) Hz and for the USAMEN 4.7 (3.6/7.2) Hz (Figure 1).

### MEN respond to ganglionic stretch and/or intraganglionic volume injection

**Figure 4:**
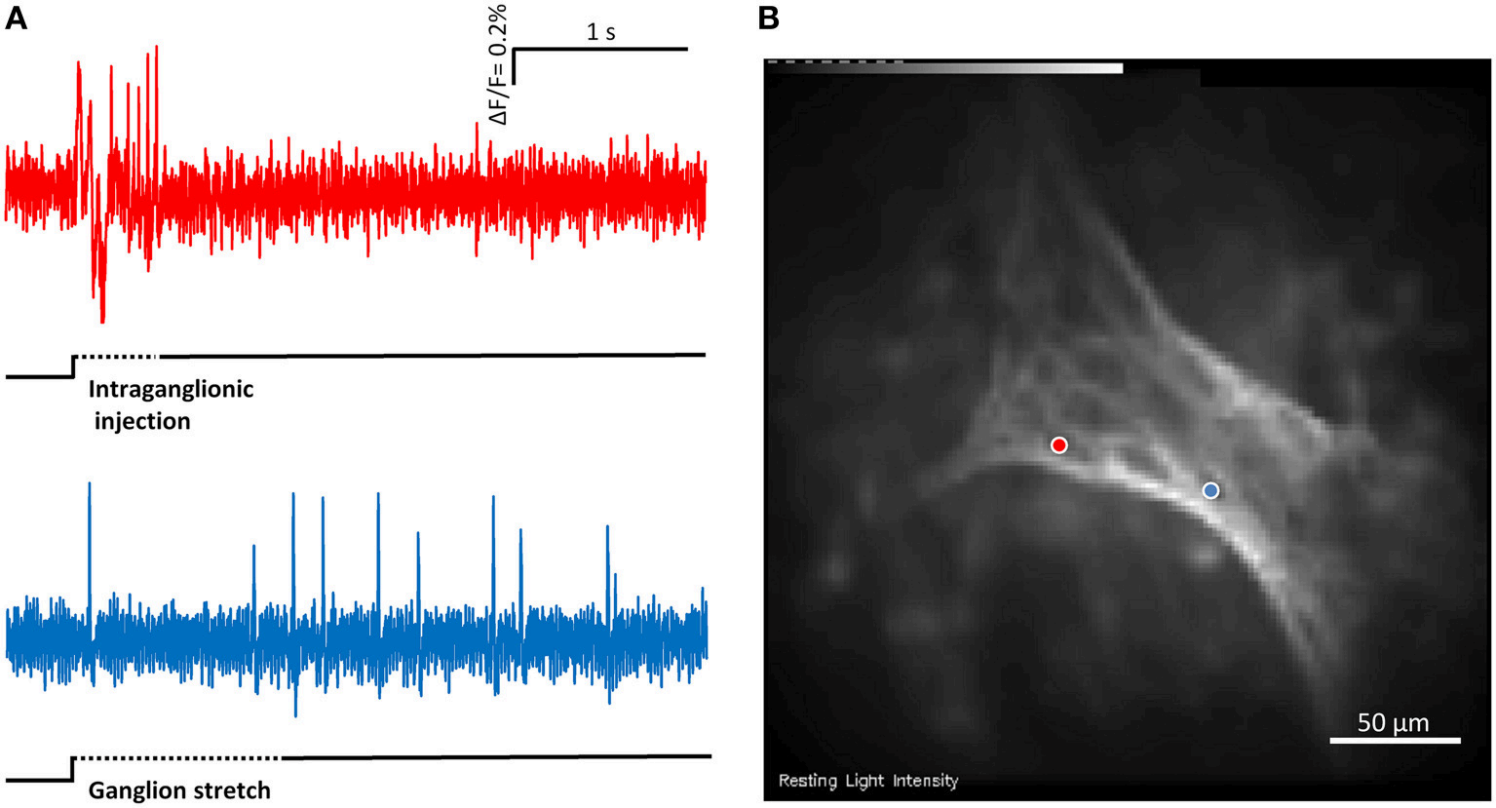
**(A)** Shows the responses of two mechanosensitive enteric neurons (MEN) from the same myenteric ganglion to compression by intraganglionic injection and tension by ganglionic stretch. The two traces in **(A)** correspond to the two neurons marked with blue and red dots in **(B)**. The compression sensitive neuron (red trace) is a rapidly adapting MEN (RAMEN) while the tension sensitive neuron (blue trace) is an ultra-slowly adapting MEN (USAMEN).

In 12 ganglia from four guinea pigs we recorded responses to stretch and intraganglionic volume injection in the same ganglion (Figure 4). The order of the stimuli was random and the recording time was 4 s.

22 ± 13% of neurons per ganglion responded to stretch, whereas 29 ± 16% responded to the intraganglionic volume injection. Of 79 mechanosensitive neurons 18 responded only to stretch, 19 only to the intraganglionic volume injection and the remaining 42 responded to both stimuli. Those neurons responding to both stimuli behaved mostly as SAMEN after intraganglionic volume injection. This dramatically changed after tissue stretch where SAMEN and USAMEN reached the same proportions. Firing of neurons responding to stretch only adapted much slower: of the 19 neurons responding only to intraganglionic volume injection 60% were RAMEN, 40% SAMEN, and 0% USAMEN. Of the 18 neurons responding exclusively to stretch 11, 56, and 33% were RAMEN, SAMEN, and USAMEN, respectively.

### Contribution of extrinsic afferents to the response of MEN

As described above, responses in some MEN depend on synaptic release, a phenomenon which we have not observed in intestinal MEN in guinea pig or mouse (Mazzuoli and Schemann, 2009, 2012). In seven ganglia from seven guinea pigs we perfused the entire tissue for 30 min with 10 μM capsaicin in order to defunctionalize extrinsic afferent fibers. This caused a slight but significant drop in the percentage of mechanosensitive neurons responding to intraganglionic volume injection from 36 (31/46) to 31 (18/39)% (*p* = 0.031). The overall firing frequency of 48 neurons was significantly decreased from 2.1 (1.0/4.1) to 0.5 (0.0/2.2) Hz (*p* < 0.001). The firing behavior did not change after capsaicin application as 61% of the neurons were RAMEN, 30% SAMEN, and 9% USAMEN.

In 12 ganglia from four guinea pigs, we perfused the tissue with the TRPV1 antagonist capsazepine. We observed a significant reduction in the number of MEN from 35 (20/51) to 28 (14/44)%. The overall spike frequency from 100 MEN was decreased from 1.7 (1.1/3.8) to 1.1 (0.1/3.3) Hz (*p* = 0.001). After TRPV1 blockade the proportion of RAMEN, SAMEN and USAMEN remained with 73, 23, and 4%, respectively, unchanged.

**Figure 5:**
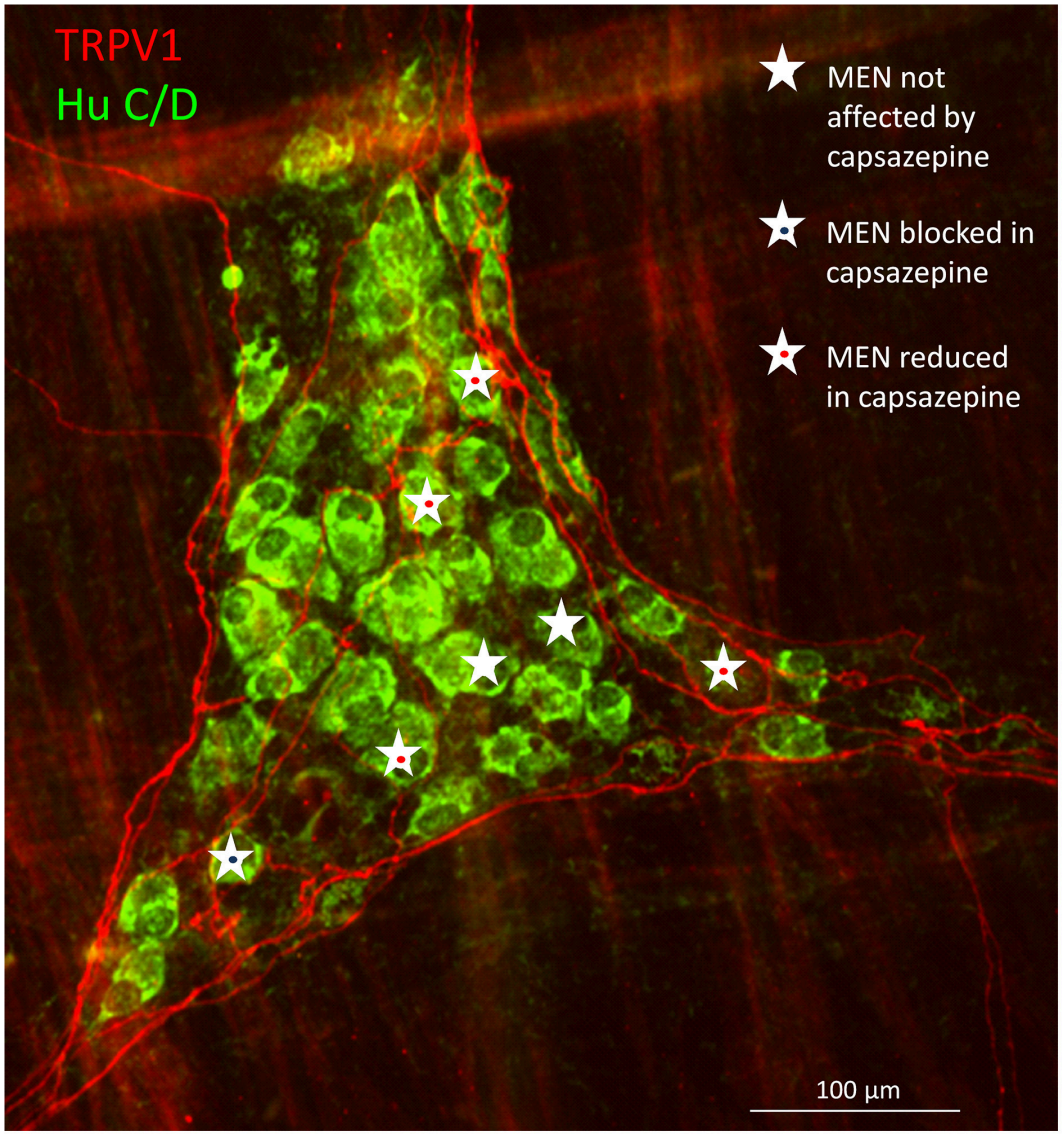
**Immunohistochemical staining of a myenteric gastric ganglion labeled by the pan-neuronal marker Hu C/D in green, transient potential receptor vanilloid (TRPV1) positive extrinsic afferent nerve terminals in red**. All mechanosensitive enteric neurons (MEN) in this ganglion are marked with white stars. The responses in MEN in close proximity to TRPV1 positive fibers were reduced or blocked by the TRPV1 antagonist capsazepine (see symbols).

Post-experimental immunohistochemical staining for TRPV1 revealed proximity of the TRPV1 positive nerve fibers with MEN which responses were affected by capsazepine (Figure 5).

Extrinsic TRPV1 expressing afferents release SP. In four ganglia from four guinea pigs we therefore tested whether the NK3 antagonist SR142801 would mimic the effects of capsaicin and capsazepine. Although the number of MEN remained constant (27 (22/36) vs. 22 (12/27)%) we found a significant decrease in overall spike frequency (*n* = 35) from 1.9 (1.1/5.2) to 1.1 (0.0/2.6) Hz (*p* = 0.003). The firing pattern of the mechanosensitive neurons after SR142801 perfusion revealed 61% RAMEN, 39% SAMEN, and no USAMEN.

### Immunohistochemistry

**Figure 6:**
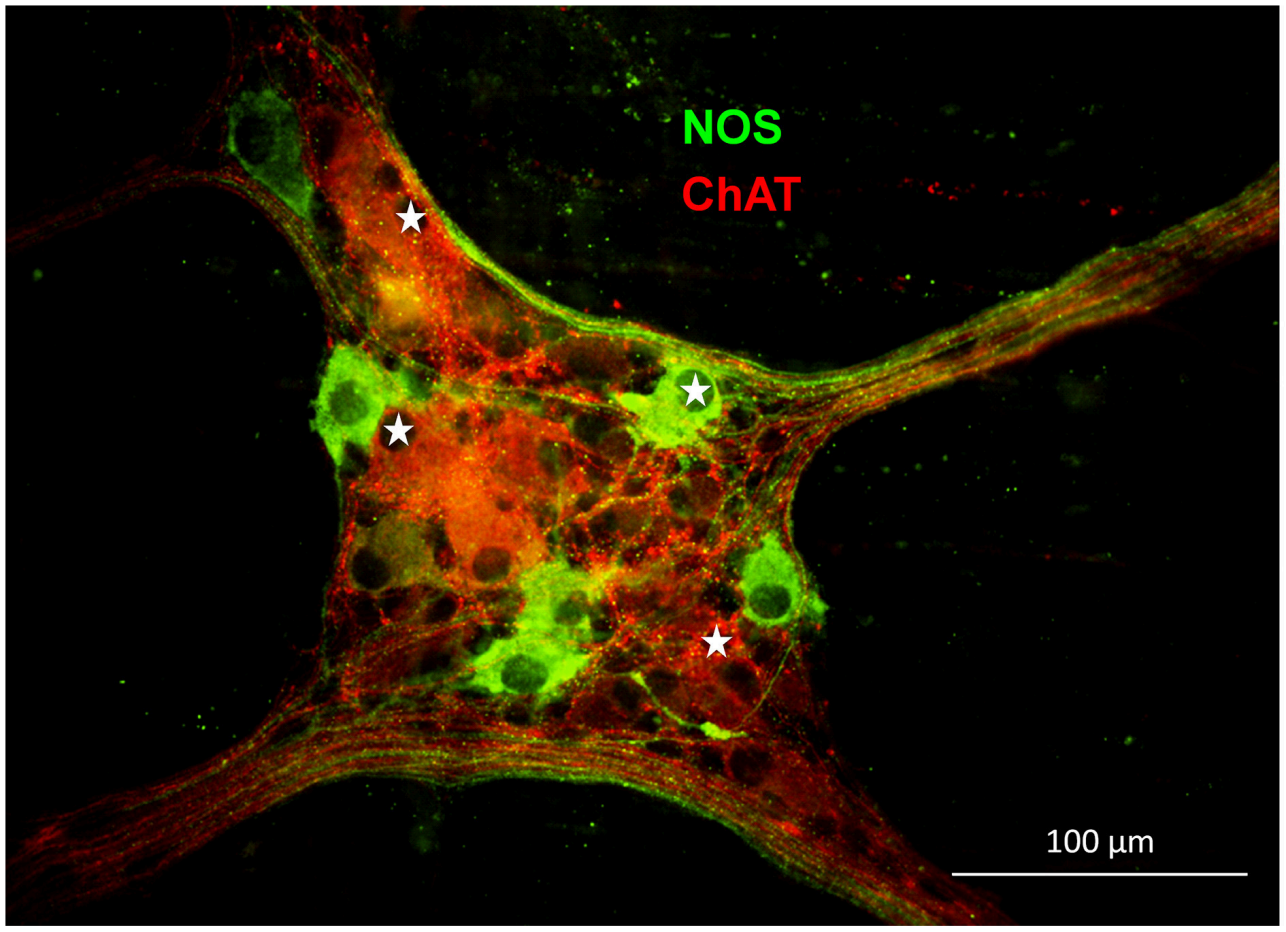
**Gastric mechanosensitive enteric neurons (MEN, marked with white stars) have nitrergic (nitric oxide synthase (NOS) positive in green) or cholinergic phenotypes (choline acetyltransferase (ChAT) positive in red)**.

In the gastric myenteric plexus 67% of myenteric neurons were ChAT-immunoreactive (IR) while 29% were NOS-IR (Schemann et al., 1995). Based on tracing experiments these were classified as motor- or interneurons (Michel et al., 1997, 2000; Brookes et al., 1998; Reiche et al., 1998; Reiche and Schemann, 1998, 1999; Pfannkuche et al., 1998a,b; Schemann et al., 2001). We found that 55% of the mechanosensitive neurons were ChAT-IR and 45% were NOS-IR (22 ganglia from five guinea pigs) (Figure 6). RAMEN were mostly ChAT-IR (56%) while 50% of the SAMEN where NOS-IR. Of 14 stretch sensitive SAMEN 64% were NOS-IR.

In the gastric corpus 12% of the myenteric neurons are Calb-IR and are considered to be interneurons because some of their processes innerve other enteric neurons (Reiche et al., 1999). Of 71 MEN (18 ganglia from three guinea pigs) only one was Calb-IR.

## Discussion

With the present work we described for the first time mechanosensitive neurons in the gastric myenteric plexus. The proportion of these neurons, transmitter coding and strong fast excitatory synaptic input was similar to those MEN previously identified by us in other intestinal regions of the guinea pig, human and mouse (Mazzuoli and Schemann, 2009, 2012; Kugler et al., 2015). Mechanosensitive responses were reproducible and stimulus strength related. Firing adapted mostly rapidly or slowly after deformation by compressive or tensile stress, respectively. Rapid adaptation was not the result of mechanoreceptor desensitization because repeated intraganglionic volume injections, each of which caused an additional deformation, evoked spike discharge. This must mean that RAMEN reset during sustained deformation and very likely do not encode the absolute degree of deformation. About half of all mechanosensitive gastric neurons responded to ganglionic stretch and intraganglionic injection suggesting that they are multimodal. Stretch and intraganglionic volume injection represent two different stimulus modalities with the stretch primarily mimicking tensile stress as also revealed by an increased ganglionic area. Intraganglionic volume injection did not cause changes in ganglionic area and evoked a mix of compressive and shear forces. However, we believe that compression is the most important stimulus modality for two reasons: firstly, flow induced shear forces had negligible effects on primary cultured MEN (own unpublished results); second, parallel experiments using von Frey hair probing (a recognized compressive stimulus) and intraganglionic injection showed that the same neurons responded to both stimuli. None of the stimulus modalities tested inhibited spike discharge because firing in spontaneously active neurons was not inhibited.

MEN firing pattern was different after application of compressive or tensile stress. Tensile stress was associated with much slower adaptation than compressive stress. After compression induced by volume injection we observed only 2% USAMEN whereas this firing behavior occurred in 44% of tension-sensitive neurons. The finding that some gastric MEN behave as USAMEN and encode sustained tension is probably linked to the prominent importance of volume adaptation which requires enteric reflex activity (Jahnberg et al., 1975). Increase in gastric volume is associated with active muscle relaxation without increase in intragastric pressure or muscle tension (Paton and Vane, 1963; Azpiroz and Malagelada, 1984, 1985; Desai et al., 1991; Hennig et al., 1997).

Tension-sensitive neurons have also been described in the colon and esophageal myenteric plexus where they also function as interneurons and inhibitory motor neurons, respectively, reinforcing the concept of multifunctionality of MEN (Spencer and Smith, 2004; Schemann and Mazzuoli, 2010; Dong et al., 2015). Similar to tension-sensitive MEN, mechanosensitive colonic interneurons appeared also to have a slowly or ultra-slowly adaptation pattern of action potential discharge (Spencer and Smith, 2004).

Compression by intraganglionic volume injection identified 27% of all neurons as mechanosensitive. This proportion was not different from the guinea pig ileum (Mazzuoli and Schemann, 2009) (*P* = 0.204), suggesting a similar pool of neurons in each gut region responsible for detecting and reacting to compressive forces. In both regions, RAMEN prevailed but the distribution of RAMEN, SAMEN, and USAMEN was nevertheless different between the two regions [Ileum 60/36/4%, gastric corpus 73/25/2% (p = 0.032)].

Mechanosensitive responses in RAMEN were reproducible after back-to-back intraganglionic injection which every time imposed a compressive force on top of a sustained deformation. These data led us to conclude that these mechanoreceptors did not desensitize but rapidly reset. This property is best known for cardiovascular mechanoreceptors (Xavier-Neto et al., 1996). In the gastrointestinal sensory physiology a resetting mechanism has been also postulated for the IGLEs in that spikes generated by one IGLE might invade other IGLEs antidromically and reset their discharge (Zagorodnyuk and Brookes, 2000; Lynn et al., 2003).

We found 27% tension sensitive neurons with about 50% also responding to compression by intraganglionic volume injection. The change in ganglionic area was about ~3% which is much smaller than the 95% increase in ganglionic area described in a distended ileal segment (Gabella and Trigg, 1984). Although some gastric myenteric neurons responded to both stimulus modalities, a large proportion appeared to be activated either by compression or stretch. The preparation was stretched during mounting and remained stretched. It may seem surprising to encounter a relatively small proportion of spontaneously active neurons. Some of them were likely stretch sensitive USAMEN as their activity increased with further deformation. Others, in particular those that did not respond to deformation might be motor neurons which were not sensitive to mechanical stimulation. As mentioned above many MEN rapidly reset and become silent during sustained deformation.

All MEN received fast EPSPs indicating that their activity may be synaptically modulated. This was not unusual and in this respect MEN resembled primary afferents neurons of the mesencephalic ganglion of the trigeminal nerve receiving synaptic inputs have been described (Verdier et al., 2004).

We have shown previously that primary cultured MEN and MEN in freshly dissected preparations responded directly to the mechanical deformation (Kugler et al., 2015; Mazzuoli and Schemann, 2009). However, in intact tissue preparation a number of mechanosensitive cells may contribute to the responses in MEN. A most likely candidate is enteric glia which would be mechanically stimulated during ganglionic deformation. Fast rise in intracellular Ca^++^ was indeed described in enteric glia after mechanical stimulation (Zhang et al., 2003). This response was significantly decreased by the phospholipase C inhibitor U73122. In our experiments U73122 did not alter the response in MEN at all. We therefore conclude that under our conditions glia has no influence.

A small proportion of MEN still responded when TTX was perfused. This was due to blockade of somal spikes because action potentials in myenteric neurons of the gastric corpus evoked by intracellular current injection were abolished by TTX (Schemann and Wood, 1989a). It is likely that the remaining spikes were carried by TTX-insensitive sodium channels because Ca-spikes were not recorded in myenteric neurons of the gastric corpus, except after blockade of tetraethylammonium sensitive potassium channels (Schemann and Wood, 1989a). Although speculative, we suggest that mechanical deformation opened TTX-insensitive sodium channels which must be voltage-independent.

Blockade of synaptic transmission, as well as defunctionalization of extrinsic afferents by long term capsaicin application resulted in a decreased number of neurons responding to deformation. This is different from the results in MEN of guinea pig ileal myenteric plexus where neither synaptic blockade nor capsaicin application affected responsiveness of mechanosensitive neurons (Mazzuoli and Schemann, 2009). The most likely explanation is the rich innervation of the stomach by extrinsic nerve fibers many of which terminate in the myenteric plexus encircling myenteric neurons. Some of them may be vagal afferents. Moreover, spinal afferent endings similar to the intraganglionic varicose endings (IGVEs) recently described in the colon (Spencer et al., 2014) have been identified in myenteric ganglia of the stomach (personal communication of Prof. Nick Spencer). Some of the extrinsic fibers are efferent nerves which are able to activate gastric myenteric neurons (Schemann and Grundy, 1992). Capsaicin sensitive TRPV1 receptors are only expressed on extrinsic fibers and not on myenteric neurons (Ward et al., 2003; and personal observation). With the present study we provided evidence that ganglionic deformation activated both the peripheral endings of extrinsic as well as intrinsic MEN and furthermore that capsaicin sensitive afferents influence the responsiveness of MEN. This very likely involved TRPV1 because responsiveness of MEN was also reduced by the TRPV1 antagonist capsazepine. The TRPV1 expressing intraganglionic endings would function as axon collaterals of vagal or spinal afferents. Distension evoked response of jejunal extrinsic afferents were also capsazepine sensitive (Rong et al., 2004). However, it remains open whether TRPV1 is directly mechanosensitive or whether the response depends on mechanosensors physically linked to TRPV1. At least part of the extrinsic to intrinsic nerve signaling involves tachykinins, because the NK3 antagonist reduced the firing of MEN. This mechanism is best described by the classical concept of axon collateral. Our findings however do not rule out involvement of other non-TRPV1 expressing extrinsic afferents.

Calbindin was found to be a quite reliable marker of AH-IPANs in the guinea pig ileum. It was not a good marker for MEN in the gastric corpus, as only 1% of the mechanosensitive neurons were positive for this calcium binding protein. This agrees with our previous results in the guinea pig ileum where calbindin was expressed by only a small proportion of MEN (Mazzuoli and Schemann, 2009). Compared to the small intestine, about twice as many gastric MEN had a nitrergic phenotype which more or less reflected the different proportions of nitregic neurons in the two regions (Mazzuoli and Schemann, 2009). This nitrergic MEN population was disproportionally larger in tension-sensitive SAMEN (64%). The functional relevance of this finding may be linked to the nerve-dependent, nitric oxide mediated muscle relaxation during gastric volume accommodation (Jahnberg et al., 1975; Desai et al., 1991). This relaxation is present over a long time and therefore requires ongoing firing of NOS-IR neurons. In the small intestine such a reflex is not as prominent and there is not such a need for NOS-IR mechanosensitive neurons. A similar function was recently proposed for tension-sensitive inhibitory motor neurons in the myenteric plexus of the esophagus (Dong et al., 2015).

In summary we identified in the guinea pig gastric corpus a population of cholinergic and nitrergic MEN which responded to compressive and/or tensile forces. While compression evoked rapid adaptation of firing, spike discharge after stretch was more sustained and adapted slowly or even ultra-slowly. Compared with the small intestine, a major difference with gastric MEN was that extrinsic afferents appeared to contribute to their mechanosensitivity. With this study we can also conclude that multifunctional MEN with comparable properties are present throughout the gut.

### Conflict of interest statement

The authors declare that the research was conducted in the absence of any commercial or financial relationships that could be construed as a potential conflict of interest.

## Abbreviations

AH: after-spike hyperpolarization
AI: adaptation index
Calb: calbindin
CCD: charge coupled device
ChAT: choline acetyltransferase
ENS: enteric nervous system
IGLEs: intraganglionic laminar endings
IMA: intramuscular arrays
IPAN: intrinsic primary afferent neuron
MEN: mechanosensitive enteric neurons
NK: neurokinin
NOS: nitric oxide synthase
RAMEN: rapidly adapting mechanosensitive enteric neurons
SAMEN: slowly adapting mechanosensitive enteric neurons
SP: substance P
TRPV: transient receptor potential vanilloid
USAMEN: ultra-slowly adapting mechanosensitive enteric neurons.

## References

[B1] Azpiroz F. Malagelada J. R. (1984). Pressure activity patterns in the canine proximal stomach: response to distension. Am. J. Physiol. 247, G265–G272. 6476116 10.1152/ajpgi.1984.247.3.G265

[B2] Azpiroz F. Malagelada J. R. (1985). Physiological variations in canine gastric tone measured by an electronic barostat. Am. J. Physiol. 248, G229–G237. 3970203 10.1152/ajpgi.1985.248.2.G229

[B3] Berthoud H. R. Powley T. L. (1992). Vagal afferent innervation of the rat fundic stomach: morphological characterization of the gastric tension receptor. J. Comp. Neurol. 319, 261–276. 10.1002/cne.903190206 1522247

[B4] Brookes S. J. Hennig G. Schemann M. (1998). Identification of motor neurons to the circular muscle of the guinea pig gastric corpus. J. Comp. Neurol. 397, 268–280. 9658288 10.1002/(sici)1096-9861(19980727)397:2<268::aid-cne8>3.0.co;2-z

[B5] Desai K. M. Zembowicz A. Sessa W. C. Vane J. R. (1991). Nitroxergic nerves mediate vagally induced relaxation in the isolated stomach of the guinea pig. Proc. Natl. Acad. Sci. U.S.A. 88, 11490–11494. 10.1073/pnas.88.24.11490 1684865 PMC53161

[B6] Dong H. Jiang Y. Dong J. Mittal R. K. (2015). Inhibitory motor neurons of the esophageal myenteric plexus are mechanosensitive. Am. J. Physiol. Cell Physiol. 308, C405–C413. 10.1152/ajpcell.00159.2014 25540174

[B7] Gabella G. Trigg P. (1984). Size of neurons and glial cells in the enteric ganglia of mice, guinea-pigs, rabbits and sheep. J. Neurocytol. 13, 49–71. 10.1007/BF01148318 6707713

[B8] Hennig G. W. Brookes S. J. Costa M. (1997). Excitatory and inhibitory motor reflexes in the isolated guinea-pig stomach. J. Physiol. 501(Pt 1), 197–212. 10.1111/j.1469-7793.1997.197bo.x 9175003 PMC1159513

[B9] Jahnberg T. Martinson J. Hultén L. Fasth S. (1975). Dynamic gastric response to expansion before and after vagotomy. Scand. J. Gastroenterol. 10, 593–598. 1179152

[B10] Kugler E. M. Michel K. Zeller F. Demir I. E. Ceyan G. O. Schemann M.. (2015). Mechanical stress activates neurites and somata of myenteric neurons. Front. Cell. Neurosci. 9:342. 10.3389/fncel.2015.00342 26441520 PMC4569744

[B11] Kummer W. Fischer A. Mundel P. Mayer B. Hoba B. Philippin B.. (1992). Nitric oxide synthase in VIP-containing vasodilator nerve fibres in the guinea-pig. Neuroreport 3, 653–655. 10.1097/00001756-199207000-00028 1384769

[B12] Li Z. S. Furness J. B. (1998). Immunohistochemical localisation of cholinergic markers in putative intrinsic primary afferent neurons of the guinea-pig small intestine. Cell Tissue Res. 294, 35–43. 10.1007/s004410051154 9724454

[B13] Lynn P. A. Olsson C. Zagorodnyuk V. Costa M. Brookes S. J. H. (2003). Rectal intraganglionic laminar endings are transduction sites of extrinsic mechanoreceptors in the guinea pig rectum. Gastroenterology 125, 786–794. 10.1016/S0016-5085(03)01050-3 12949724

[B14] Mawe G. M. Schemann M. Wood J. D. Gershon M. D. (1989). Immunocytochemical analysis of potential neurotransmitters present in the myenteric plexus and muscular layers of the corpus of the guinea pig stomach. Anat. Rec. 224, 431–442. 10.1002/ar.1092240312 2476950

[B15] Mazzuoli G. Schemann M. (2009). Multifunctional rapidly adapting mechanosensitive enteric neurons (RAMEN) in the myenteric plexus of the guinea pig ileum. J. Physiol. 587, 4681–4694. 10.1113/jphysiol.2009.177105 19703967 PMC2768021

[B16] Mazzuoli G. Schemann M. (2012). Mechanosensitive enteric neurons in the myenteric plexus of the mouse intestine. PLoS ONE 7:e39887. 10.1371/journal.pone.0039887 22768317 PMC3388088

[B17] Mazzuoli-Weber G. Schemann M. (2015). Mechanosensitivity in the enteric nervous system. Rev. Front. Cell. Neurosci. 9:408 10.3389/fncel.2015.00408 PMC4602087 26528136

[B18] Michel K. Michaelis M. Mazzuoli G. Mueller K. Vanden Berghe P. Schemann M. (2011). Fast calcium and voltage-sensitive dye imaging in enteric neurones reveal calcium peaks associated with single action potential discharge. J. Physiol. 589, 5941–5947. 10.1113/jphysiol.2011.219550 22041184 PMC3286677

[B19] Michel K. Reiche D. Schemann M. (2000). Projections and neurochemical coding of motor neurones to the circular and longitudinal muscle of the guinea pig gastric corpus. Pflüg. Arch. Eur. J. Physiol. 440, 393–408. 10.1007/s004240000299 10954325

[B20] Michel K. Sann H. Schaaf C. Schemann M. (1997). Subpopulations of gastric myenteric neurons are differentially activated via distinct serotonin receptors: projection, neurochemical coding, and functional implications. J. Neurosci. 17, 8009–8017. 9315919 10.1523/JNEUROSCI.17-20-08009.1997 PMC6793905

[B21] Neunlist M. Peters S. Schemann M. (1999). Multisite optical recording of excitability in the enteric nervous system. Neurogastroenterol. Motil. 11, 393–402. 10.1046/j.1365-2982.1999.00163.x 10520170

[B22] Paton W. D. Vane J. R. (1963). Analysis of the responses of the isolated stomach to electrical stimulation and to drugs. J. Physiol. 165, 10–46. 10.1113/jphysiol.1963.sp007040 13941859 PMC1359254

[B23] Petkov G. V. Boev K. K. (1999). Control of the phasic and tonic contractions of guinea pig stomach by a ryanodine-sensitive Ca^2+^ store. Eur. J. Pharmacol. 367, 335–341. 10.1016/S0014-2999(98)00875-9 10079009

[B24] Pfannkuche H. Reiche D. Firzlaff U. Sann H. Schemann M. (1998a). Enkephalin-immunoreactive subpopulations in the myenteric plexus of the guinea-pig fundus project primarily to the muscle and not to the mucosa. Cell Tissue Res. 294, 45–55. 10.1007/s004410051155 9724455

[B25] Pfannkuche H. Reiche D. Sann H. Schemann M. (1998b). Different subpopulations of cholinergic and nitrergic myenteric neurones project to mucosa and circular muscle of the guinea-pig gastric fundus. Cell Tissue Res. 292, 463–475. 10.1007/s004410051075 9582403

[B26] Reiche D. Huber K. Hoppe S. Schemann M. (2001). Neurochemically distinct myenteric neurone populations containing calbindin have specific distribution patterns around the circumference of the gastric corpus. Cell Tissue Res. 303, 319–328. 10.1007/s004410000332 11320647

[B27] Reiche D. Pfannkuche H. Michel K. Hoppe S. Schemann M. (1999). Immunohistochemical evidence for the presence of calbindin containing neurones in the myenteric plexus of the guinea-pig stomach. Neurosci. Lett. 270, 71–74. 10.1016/S0304-3940(99)00471-1 10462100

[B28] Reiche D. Pfannkuche H. Michel K. Schemann M. (1998). Structural and functional organization of the enteric nervous system in the stomach. Dtsch. Tierärztl. Wochenschr. 105, 461–465. 9932017

[B29] Reiche D. Schemann M. (1998). Ascending choline acetyltransferase and descending nitric oxide synthase immunoreactive neurones of the myenteric plexus project to the mucosa of the guinea pig gastric corpus. Neurosci. Lett. 241, 61–64. 10.1016/S0304-3940(97)00968-3 9502216

[B30] Reiche D. Schemann M. (1999). Mucosa of the guinea pig gastric corpus is innervated by myenteric neurones with specific neurochemical coding and projection preferences. J. Comp. Neurol. 410, 489–502. 10404414 10.1002/(sici)1096-9861(19990802)410:3<489::aid-cne10>3.0.co;2-s

[B31] Rong W. Hillsley K. Davis J. B. Hicks G. Winchester W. J. Grundy D. (2004). Jejunal afferent nerve sensitivity in wild-type and TRPV1 knockout mice. J. Physiol. 560(Pt 3), 867–881. 10.1113/jphysiol.2004.071746 15331673 PMC1665286

[B32] Schemann M. Grundy D. (1992). Electrophysiological identification of vagally innervated enteric neurons in guinea pig stomach. Am. J. Physiol. 263, G709–G718. 1443146 10.1152/ajpgi.1992.263.5.G709

[B33] Schemann M. Kayser H. (1991). Effects of tachykinins on myenteric neurones of the guinea-pig gastric corpus: involvement of NK-3 receptors. Pflügers. Arch. 419, 566–571. 10.1007/BF00370296 1724075

[B34] Schemann M. Mazzuoli G. (2010). Multifunctional mechanosensitive neurons in the enteric nervous system. Auton. Neurosci. 153, 21–25. 10.1016/j.autneu.2009.08.00 19744894

[B35] Schemann M. Reiche D. Michel K. (2001). Enteric pathways in the stomach. Anat. Rec. 262, 47–57. 10.1002/1097-0185(20010101)262:1<47::AID-AR1010>3.0.CO;2-1 11146428

[B36] Schemann M. Rohn M. Michel K. (2008). Motor control of the stomach. Eur. Rev. Med. Pharmacol. Sci. 12(Suppl. 1), 41–51. 18924443

[B37] Schemann M. Schaaf C. (1995). Differential projection of cholinergic and nitroxidergic neurons in the myenteric plexus of guinea pig stomach. Am. J. Physiol. 269, G186–G195. 7544534 10.1152/ajpgi.1995.269.2.G186

[B38] Schemann M. Schaaf C. Mäder M. (1995). Neurochemical coding of enteric neurons in the guinea pig stomach. J. Comp. Neurol. 353, 161–178. 10.1002/cne.903530202 7538152

[B39] Schemann M. Wood J. D. (1989a). Electrical behaviour of myenteric neurones in the gastric corpus of the guinea-pig. J. Physiol. 417, 501–518. 10.1113/jphysiol.1989.sp017815 2621607 PMC1189280

[B40] Schemann M. Wood J. D. (1989b). Synaptic behaviour of myenteric neurones in the gastric corpus of the guinea-pig. J. Physiol. 417, 519–535. 10.1113/jphysiol.1989.sp017816 2621608 PMC1189281

[B41] Schicho R. Krueger D. Zeller F. Von Weyhern C. W. H. Frieling T. Kimura H.. (2006). Hydrogen sulfide is a novel prosecretory neuromodulator in the Guinea-pig and human colon. Gastroenterology 131, 1542–1552. 10.1053/j.gastro.2006.08.035 17101327

[B42] Sharkey K. A. Williams R. G. Dockray G. J. (1984). Sensory substance P innervation of the stomach and pancreas. Demonstration of capsaicin-sensitive sensory neurons in the rat by combined immunohistochemistry and retrograde tracing. Gastroenterology 87, 914–921. 6205934

[B43] Spencer N. J. Kyloh M. Duffield M. (2014). Identification of different types of spinal afferent nerve endings that encode noxious and innocuous stimuli in the large intestine using a novel anterograde tracing technique. PLoS ONE 9:e112466. 10.1371/journal.pone.0112466 25383884 PMC4226564

[B44] Spencer N. J. Smith T. K. (2004). Mechanosensory S-neurons rather than AH-neurons appear to generate a rhythmic motor pattern in guinea-pig distal colon. J. Physiol. 558, 577–596. 10.1113/jphysiol.2004.063586 15146052 PMC1664963

[B45] Verdier D. Lund J. P. Kolta A. (2004). Synaptic inputs to trigeminal primary afferent neurons cause firing and modulate intrinsic oscillatory activity. J. Neurophysiol. 92, 2444–2455. 10.1152/jn.00279.2004 15381749

[B46] Wang F. B. Powley T. L. (2000). Topographic inventories of vagal afferents in gastrointestinal muscle. J. Comp. Neurol. 421, 302–324. 10.1002/(SICI)1096-9861(20000605)421:3<302::AID-CNE2>3.0.CO;2-N 10813789

[B47] Ward S. M. Bayguinov J. Won K.-J. Grundy D. Berthoud H. R. (2003). Distribution of the vanilloid receptor (VR1) in the gastrointestinal tract. J. Comp. Neurol. 465, 121–135. 10.1002/cne.10801 12926020

[B48] Weber E. Neunlist M. Schemann M. Frieling T. (2001). Neural components of distension-evoked secretory responses in the guinea-pig distal colon. J. Physiol. 536, 741–751. 10.1111/j.1469-7793.2001.00741.x 11691869 PMC2278890

[B49] Xavier-Neto J. Moreira E. D. Krieger E. M. (1996). Viscoelastic mechanisms of aortic baroreceptor resetting to hypotension and to hypertension. Am. J. Physiol. 271, H1407–H1415. 8897934 10.1152/ajpheart.1996.271.4.H1407

[B50] Zagorodnyuk V. P. Brookes S. J. (2000). Transduction sites of vagal mechanoreceptors in the guinea pig esophagus. J. Neurosci. 20, 6249–6255. 10934275 10.1523/JNEUROSCI.20-16-06249.2000 PMC6772604

[B51] Zagorodnyuk V. P. Chen B. N. Brookes S. J. H. (2001). Intraganglionic laminar endings are mechano-transduction sites of vagal tension receptors in the guinea-pig stomach. J. Physiol. 534, 255–268. 10.1111/j.1469-7793.2001.00255.x 11433006 PMC2278677

[B52] Zhang W. Segura B. J. Lin T. R. Hu Y. Mulholland M. W. (2003). Intercellular calcium waves in cultured enteric glia from neonatal guinea pig. Glia 42, 252–262. 10.1002/glia.10215 12673831

