# Supplementary material for: Mechanosensitive enteric neurons in the guinea pig gastric corpus
Source: Front Cell Neurosci. 2015 Nov 3;9:430. doi: 10.3389/fncel.2015.00430 (PMC4630284; doi:10.3389/fncel.2015.00430)
Supplement: Supplementary file 2 [file Video1-Legend.DOCX]

Supplementary Material

**Mechanosensitive enteric neurons in the guinea pig gastric corpus**

**Gemma Mazzuoli-Weber^1^*, Michael Schemann^1^**

^1^Human Biology, Technische Universitaet Muenchen, Freising, Germany

*** Correspondence:**

Gemma Mazzuoli-Weber

Department of Human Biology

Technische Universitaet Muenchen

Liesel-Beckmann-Strasse 4,

Freising, 85354, Germany

[gemma.mazzuoli@wzw.tum.de](mailto:gemma.mazzuoli@wzw.tum.de)

# Supplementary Data

**Movie 1**

This slow motion movie (original length is 1.71 sec) demonstrates deformation in a gastric ganglion during intraganglionic volume injection and spike discharge in 2 mechanosensitive enteric neurons (MEN). The outline of individual neurons can be seen because Di-8-ANEPPS incorporates into the membrane. The two MEN are marked with red and green arrowheads. The red one behaved as a rapidly adapting MEN (RAMEN), while the green one behaved as a slowly adapting MEN (SAMEN). The traces at the bottom correspond to the two MEN. Once the movie starts there is a running bar indicating the elapsed time. The bar is running from 0 to 1.71 sec. The blue arrow appearing on the left upper corner indicates the onset of the volume injection. With beginning of the volume injection from the left one can see the deformation of neurons. The overlay illustrates the color coded signals from the two neurons; red color indicates discharge of action potentials. Shortly after the onset of the volume injection the red neuron (RAMEN) fired a volley of action potentials (red color) stopping after 500 ms. The green neuron (SAMEN) required a longer adaptation time and fired throughout the recording period.
